# Biomimetic Venus Flytrap Structures Using Smart Composites: A Review

**DOI:** 10.3390/ma16206702

**Published:** 2023-10-16

**Authors:** Bing Wang, Yi Hou, Shuncong Zhong, Juncheng Zhu, Chenglong Guan

**Affiliations:** 1 School of Advanced Manufacturing, Fuzhou University, Fuzhou 362251, China; b.wang@fzu.edu.cn (B.W.);; 2 Fujian Provincial Key Laboratory of Terahertz Functional Devices and Intelligent Sensing, School of Mechanical Engineering and Automation, Fuzhou University, Fuzhou 350108, China; guancl@fzu.edu.cn

**Keywords:** biomimetic, Venus flytrap, smart, composite, mechanics

## Abstract

Biomimetic structures are inspired by elegant and complex architectures of natural creatures, drawing inspiration from biological structures to achieve specific functions or improve specific strength and modulus to reduce weight. In particular, the rapid closure of a Venus flytrap leaf is one of the fastest motions in plants, its biomechanics does not rely on muscle tissues to produce rapid shape-changing, which is significant for engineering applications. Composites are ubiquitous in nature and are used for biomimetic design due to their superior overall performance and programmability. Here, we focus on reviewing the most recent progress on biomimetic Venus flytrap structures based on smart composite technology. An overview of the biomechanics of Venus flytrap is first introduced, in order to reveal the underlying mechanisms. The smart composite technology was then discussed by covering mainly the principles and driving mechanics of various types of bistable composite structures, followed by research progress on the smart composite-based biomimetic flytrap structures, with a focus on the bionic strategies in terms of sensing, responding and actuation, as well as the rapid snap-trapping, aiming to enrich the diversities and reveal the fundamentals in order to further advance the multidisciplinary science and technological development into composite bionics.

## 1. Introduction

Biomimetic structures are inspired by elegant and complex architectures of natural creatures, which can be lightweight and offer combinations of mechanical properties that often surpass those of their components by orders of magnitude [1]. Biomimetic structures are often conceived to achieve specific functions, drawing inspiration from biological structures [2]. A typical example is the flexible solar array, the core part of a spacecraft power generation system, which is limited by space during transportation; whilst a deployable solar array similar to the convolvulaceae increases the stowed-to-deployed ratio [3]. For the biomimetic convolvulaceae deployable structure, petals are spirally folded around the center to form a floral bud, and then slowly open along the radial direction of the flower until full bloom. Another attraction is the fast nastic motion in nature. The ballistic projection of a chameleon’s tongue is a typical example of rapid motion due to elastic forces [4]. It involves a complex interplay between the internal organization, collagen fibrous, stress release and geometry [5]. Hummingbirds can prey on insects because their beaks have the ability to impede quickly, the high-speed capture motion cannot be explained only by direct muscle action, possibly powered by the sudden release of stored elastic energy [6]. The rapid closure of a Venus flytrap leaf is one of the fastest motions in plants, once a principal natural curvature of the leaf changes, there will be a fast snap-through phenomenon [7]. These unique biological structural mechanics have gone through a lengthy evolution, providing optimized solutions for desired functional systems. Scientists and engineers have explored and imitated for decades and continue to investigate in order to reveal the underlying mechanisms, aiming to benefit and facilitate practical engineering designs and applications for industries [8].

Composite materials have attracted growing interest for their design and manufacturing flexibilities, high specific strength and stiffness, which are superior to reducing structural weight and functional complexities [9]. Smart morphing composite technologies are developed to design and manufacture structures that can sense and respond to ambient environmental changes [10]. In particular, a bistable composite is a thin shell laminate structure, which is able to undergo repeated reversible changes between two stable shapes under external stimuli [11], analogous to rapid morphing mechanisms in nature.

The biomimetic Venus flytrap structures are found mostly by employing the morphing mechanics of smart composites. These contribute to their main characteristics and advantages in terms of (i) structural design freedom, since organisms are variable and complex in nature; (ii) simplicity and lightweight, the conventional deformable bioinspired structure connected by multiple rigid modules through motion pairs is complex and heavy, whilst the shape-changing process of a thin-shelled smart composite is similar to rapid snap-trapping of a Venus flytrap leaf, simplifying the design of biomimetic structures; (iii) energy conservation and flexibility, the smart composite structure stores shapes through binary states, the shape-changing process is reversible without additional input energy, which offers efficient and flexible energy-saving rapid morphing mechanics driven by stored elastic energy.

There have been reviews concerning the bionics of the Venus flytrap. The research progress on plant-inspired adaptive structures and materials for morphing and actuation has been reported in [2], where the Venus flytrap was introduced as one of the imitated creatures for impulsive and reversible movements. The fast active motion principle inspired by the Venus flytrap was summarized in [12] with a focus on the application to soft machine systems. Here, we focus on reviewing the most recent development in the smart composite-based biomimetic Venus flytrap structures. Firstly, we gave an overview of the biomechanics of the Venus flytrap in order to reveal the underlying mechanisms. The smart composite technology was then introduced by covering mainly the principles and driving mechanics of various types of smart composite structures, followed by the research progress on the smart composite-based biomimetic flytrap structures, with a focus on the bionic strategies in terms of sensing, responding and actuation, as well as the rapid snap-trapping, aiming to enrich the diversities and reveal the fundamentals in order to further advance the multidisciplinary science and technological development into composite bionics.

## 2. Biomechanics of Venus Flytrap

**Figure 1:**
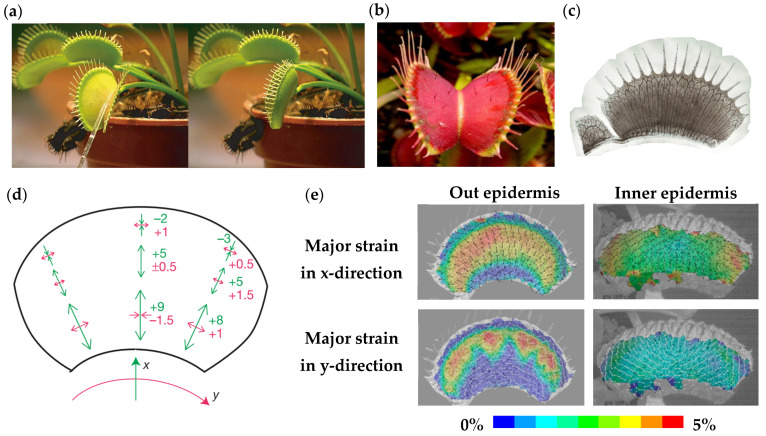
Biomechanics of Venus flytrap, showing: (**a**) open and closed status of a flytrap leaf [7]; (**b**) structural characteristics of the highly sensitive trigger hairs on the inner surface; (**c**) side view of the vascular structure; (**d**) internal stress field of a leaf following the single tissue layer model [17]; (**e**) the strain distribution of a leaf following the bi-layer model [19].

Venus flytrap is a magic insect-trapping plant that catches agile insects by the rapid closure of a pair of symmetrical shell-like leaves [13]. When the Venus flytrap is stimulated, the geometry of flytrap leaves changes from convex to concave shapes within 100 ms, see Figure 1a, similar to the snapping of a bistable composite structure [7]. The biomechanics of the Venus flytrap does not rely on muscle tissues to produce rapid shape-changing, which is significant for engineering applications.

The trigger hairs on the inner surface of the flytrap leaves are highly sensitive, see Figure 1b, they bend when sensing moving insects [14]. The sensitive cells produce initial action potential, and their structural characteristics contribute to the rapid propagation of action potential [15]. The action potential is the form of electrical signal transmission, which propagates between plant cells through the vasculature system [16]. Figure 1c shows a side view of the vascular structure within a Venus flytrap leaf. The propagation of action potential depends on the transmembrane flow of ions, with a speed of up to 10 m/s [17]. A hypothesis on the rapid trapping movement is then proposed: when the action potential is diffused in the motor tissue, a fast permeability change leads to massive leakage of ions; the rapid decrease in internal osmotic pressure causes the Venus flytrap leaves to close rapidly [18]. Thus, cell dimensional changes are amplified in the movement of attached organs.

Osmotic driven (active water transport) is crucial for active motion but cannot explain the rapid trapping process of the Venus flytrap. An important reason is that the snap-trap is not caused by the bending of the entire leaves, but a snap-buckling instability through active control [7]. The geometry of the doubly-curved leaf is bistable, which provides mechanisms through elastic energy storage and release. It is the passive motion of the trap, and manifested by the release of prestress. The internal stress field of flytrap leaves is considered to follow the geometry of the leaves but is locally orthotropic. Figure 1d shows the internal stress field and distribution [17]. The variation of curvature in the *x*-direction caused by the biochemical reaction is much larger than that in the *y*-direction, which is considered to be the actuating factor of the snapping. To dig further into the triggering mechanics of the passive motion at a macroscopic scale, a more detailed bi-layer model has been developed to simulate the internal stress/strain field, see Figure 1e. It is confirmed that the trapping process is the interaction between the shrinking/swelling processes of the various tissue layers, as well as the release of stored elastic strain energy [19].

Therefore, the biomechanics of the Venus flytrap consists of two mechanisms: (i) the biochemical reaction through changes in osmotic pressure as explicit sensing and trigger actuation; (ii) the bistable shape transition as the implicit fast trapping motion. Thus, the progress and development of smart composite-based biomimetic flytrap structures are also concentrated on mimicking the smart sensing and actuation strategies, as well as the rapid snap-trapping phenomenon.

## 3. Smart Composite Technology

Smart materials and composite technology are derived from the 1980s. It refers to a material or structural system that is capable of sensing external changes, and responding to stimuli by changing their specific properties, geometric configurations, etc. [20]. They have been widely applied to medical devices, flexible electronics, as well as aerospace engineering [21]. A smart material can transform from a temporary configuration to its original configuration in response to external stimuli: this is well-known as the shape memory effect. Especially, shape-memory materials have attracted great interest due to their unique shape-memory effects, these mainly include shape-memory alloys (SMAs) [22], as well as shape-memory polymers (SMPs) [23]. SMAs are usually low in recovery rate, whilst SMPs are high in recovery rate, low in density, and possess large shape morphing ability that can be beneficial for intelligent morphing designs [24]. Commonly used thermoplastic SMPs include polylactic acid (PLA), polycaprolactone (PCL), polyester (PE), polyurethane (PU), etc. [25]; shape memory effects have also been identified in thermoset polymers such as epoxy and photo-reactive resins [26,27], as well as in hydrogels [28].

Smart composites are developed based on smart polymers with improved mechanical performance. Depending on the specific material and structure employed to produce a smart composite, it can be driven by heat [29], light [30], magnetism [31], electricity [32], or water/moisture [33], etc. They are superior in terms of diversity in smart driving strategy, flexibility in structural programming, highly recoverable ability, large deformation capability, as well as good biocompatibility [34]. Therefore, they are ideal for applications to sensors and actuators within a biomimetic flytrap to sense and initiate shape-changing actuation.

A prelude of smart composite structures also includes those that are bistable, enabling elastic strain energy-based rapid morphing of the biomimetic flytrap. In producing a composite, it is known that thermal residual stress would be generated during cooling, which may lead to deflection, local buckling, and even premature failure of a composite structure in service [35,36]. Although they are usually detrimental, they can also be beneficial to produce bistable or multistable composite structures. Hyer [37] first discovered the bistability from producing unsymmetric thin laminates, where orthogonal deflections result in a bistable structure, and the Classical Laminate Theory (CLT) was found not valid to predict the saddle shapes. It was further expanded by considering the geometric nonlinearity of the stress-strain relationship and analyzing the size effects on bistability [38,39].

**Figure 2:**
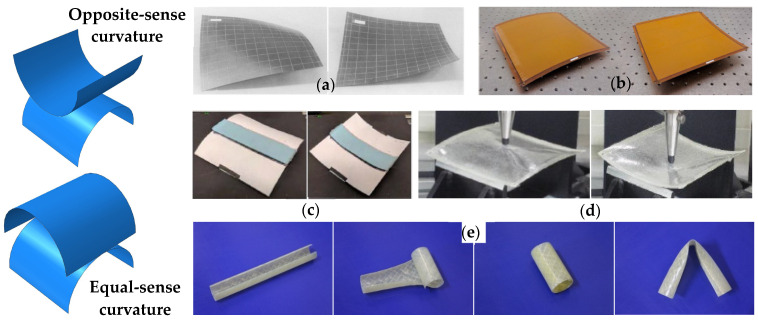
Bistable composite structures developed by employing (**a**) thermal residual stress [42]; (**b**) piezoelectric actuation [43]; (**c**) continuous elastic fiber prestressing [45]; (**d**) continuous viscoelastic fiber prestressing [47]; (**e**) geometrical curvature effects [49].

There are mainly two types of bistable composite structures considering the curvature directions of the two stable shapes, namely (i) opposite-sense bistable (OSB) structure, and (ii) equal-sense bistable (ESB) structure, see Figure 2. Although composites have also been used to explore bistable twisted structures [40,41], these are out of the scope of the current focus. It is reported that for (i), OSB structures can be produced by adjusting in-plane stress levels, these are mainly achieved by employing thermal residual stress [37,42], piezoelectric actuation [43,44], continuous elastic fiber prestressing [45,46], as well as continuous viscoelastic fiber prestressing [11,47]; whilst for (ii), ESB structures are mainly produced by exploring the geometric curvature effects [48], also commonly known as composite tape-springs [49,50].

The thermal residual stress-induced OSB structure is the most established, which is produced by employing unsymmetric composite layups. A typical example is shown in Figure 2a. Owning to a mismatch in thermal expansion between fiber and matrix materials, thermal residual stresses are developed during cooling, leading to unsymmetric in-plane stresses, resulting in out-of-plane deflections in order to generate bistability. However, these are found sensitive to temperature and humidity conditions, and difficult to control precisely [51,52]. For the piezoelectric actuation-based OSB structure, in-plane stress is altered by using piezoelectric strips which are responsive to temperature changes and controlled through voltage [43], see Figure 2b. The continuous fiber prestressing techniques employ elastic (see Figure 2c) or viscoelastic (see Figure 2d) recovery within a composite structure, which would introduce compressive stresses and interact with the intrinsic thermal residual stress in order to induce out-of-plane deflections [53,54]. As for ESB structure, a composite tape-spring structure explores the positive Gaussian curvature effects, see Figure 2e, the governing factors of its bistability depend on the material constitutive behavior, initial geometric proportions, as well as the geometrically non-linear structural behavior [55].

## 4. Biomimetic Venus Flytrap Structures

Plants that harvest energy from nature through photosynthesis accomplish perception and response to changing conditions without brain control. They provide possibilities for constructing advanced biomimetic structures that rely on adaptive material systems without control centers. The sense and response of a material system to changing environmental conditions are related to the corresponding biological reaction mechanisms. Therefore, plant-inspired biomimetic structures have focused on the achievement of plant functional principles, rather than simply imitating the behavior of plant movement in past decades [12]. These biomimetic structures, which appeared closely to the biological model, are to achieve self-growth and self-repair and respond correctly when sensing changes in ambient conditions. Since these strategies are still in their infancy stage, the existing Venus flytrap biomimetic structures are still not capable of self-growing and self-repairing. Therefore, the main biomimetic characteristics in terms of sensing, and actuation, as well as the rapid snap-trapping phenomenon of the biomimetic flytrap structures are mainly summarized here, with a focus on exploring the smart composite technology.

### 4.1. Electric-Driven Sensing and Actuation

The function of sensing for biomimetic flytrap structure is usually achieved by integrating smart material-based sensors. To date, the sensing characteristics of ionic polymer metal composites (IPMCs) are the most popular to mimic the trigger hairs of flytrap leaves. IPMC is an intelligent material superior in terms of bidirectional motion, fast response, and low driving voltage. When it is bent by an external force, the solvent is replaced, and the resulting charge polarisation generates a voltage on both sides of the material. Based on this acting principle, the IPMC has been applied to mimetic the trigger hairs, i.e., bending sensor. Under the influence of an external electric field, the redistribution of water molecules in IPMC leads to its bending deformation, involving a series of energy conversions, including electrical energy, chemical energy and mechanical energy.

**Figure 3:**
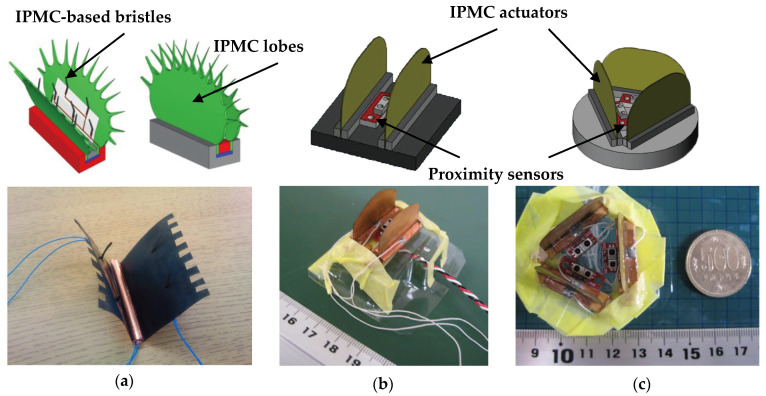
Electric-driven ionic polymer metal composite-based biomimetic flytrap structure, showing (**a**) a small biomimetic flytrap-based robot [58]; (**b**,**c**) proximity sensors are applied to imitate the trigger hairs to sense adjacent approaching objects [57].

The sensing characteristics and mechanical bending of an IPMC system are similar to the sensing and actuation of the flytrap. Figure 3a shows a small biomimetic flytrap-based robot employing the IPMC-based sensor and actuator [56]. The IPMC-based bristles initiate signals via an amplifying circuit when subjected to bending, and trigger the actuation circuit of the IPMC lobes, which are then bent rapidly and close within nearly 0.3 s. Although the sensing characteristics of IPMC related to internal ion transfers are similar to those of a real flytrap, the output of IPMC brush as a sensor within a biomimetic structure is rather weak and unstable, its bending is also limited. The infrared proximity sensors were then developed to replace the IPMC bristles, Figure 3b,c show typical examples [57]: in order to further improve the capability in sensing, number of proximity sensors can be increased; to obtain maximal shape-changing, three IPMC lobes were used to reduce gaps. 

### 4.2. Water-Driven Sensing and Actuation

**Figure 4:**
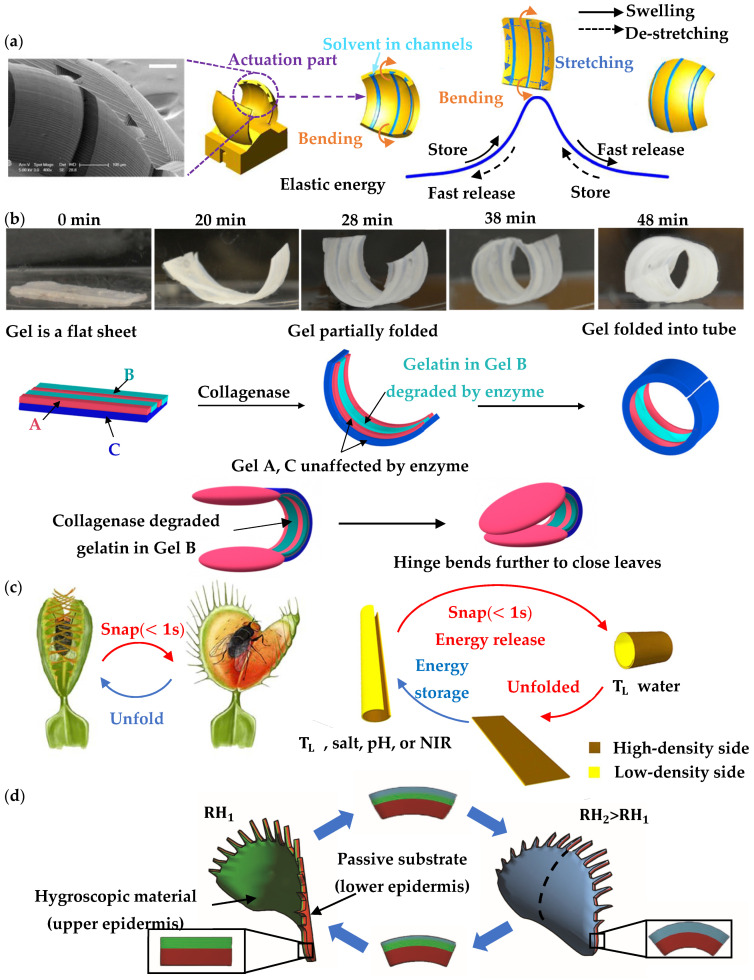
Water-driven hydrogel-based biomimetic flytrap structure, showing (**a**) a hydrogel-based doubly bending structure incorporating elastic instability [59]; (**b**) a bi-layered biomimetic flytrap based on a gel consisting of a mixture of three different components that is triggered by using enzyme [60]; (**c**) temperature response process of a rGO/PDMAEMA composite hydrogel sheet-based snap-trap structure [61]; (**d**) a pure humidity-responsive artificial flytrap leaf based on hygroscopic bistability [62].

The sensing function of biomimetic structures is often achieved by using stimulus-responsive materials, which are sensitive to changes in environmental conditions and deformed accordingly. A notable feature of hydrogels is that their volume changes during a wide range of environmental conditions. However, the leaves of a flytrap can quickly close within a tenth of a second to catch insects, significantly different from the usual slow shape transition of hydrogels. Therefore, the ingenious combination of sensing external stimuli and improving responsive speed needs to be implemented in hydrogel-based biomimetic designs. Figure 4a shows a hydrogel-based doubly bending structure incorporating elastic instability [59]. The hydrogel leaf protrudes outward, and three microfluidic channels are embedded on its inner surface for solvent transportation. The expansion of hydrogel is controlled in the same way as in the deformation of a flytrap leaf, only the curvature in one direction is actively manipulated by changes in solvent through the microfluidic channels, and the curvature in the other direction remains passive. During the swelling of the hydrogel, bending–stretching coupling of the doubly-curved geometry stores elastic strain energy along the axis. With further expansion, the stored elastic potential energy is released instantaneously after passing through the energy barrier, leading to the snap-buckling of the hydrogel leaf. During the drying or de-swelling process, reverse movements occur and quickly return to the original shape of the leaf. The whole shape-changing cycle can be controlled within 5 s.

It is worth noting that soft materials in nature are inhomogeneous. They have multiple functional regions with different chemical and mechanical compositions. Therefore, the composition and distribution of different materials could also be learned for biomimetic response structural systems. Figure 4b shows a bi-layered biomimetic flytrap, which is designed based on a mixture of three different gels that is triggered by an enzyme in the solution in order to change shapes [60]. The gels are constructed into a bilayer structure, which consists of two layers: a gel A/B layer is sandwiched above a layer of gel C. Two elliptical leaves made of gel A are connected by a hinge made of a mixed gel. Initially, when the biomimetic flytrap is placed in water, the gel C layer swells more than the gel A/B layer. Despite the mismatch in swelling speed, the hinge remains flat since the gel A/B layer is produced to be stiffer. In addition to the collagenase enzyme in the water, it cleaves the gelatin chains in gel B, which then reduces the stiffness of the A/B layer. The swollen gel C layer is now able to fold over the A/B layer, leading to the hinge being transformed into a specific shape. The reaction time for the leaf to be fully folded is about 50 min for 50 U/mL of enzyme. It should be noted that it is necessary to include energy storage and release mechanisms in order to improve the response period.

Another concept of snap-trap based on the rGO/PDMAEMA composite hydrogel sheet is shown in Figure 4c, associated with the temperature response process [61]. The composite hydrogel sheet has a double gradient along the thickness direction, i.e., chain density and cross-linking density gradient, which is able to accumulate elastic energy and release the stored energy rapidly through ultrafast snapping deformation. The composite gel is flat when immersed in 20 °C water. When immersed in 60 °C water, the composite sheet is bent along the longitudinal axis to the higher gradient side and transferred into a tubular structure, with stored energy. When replaced with the 20 °C water, the hydrogel sheet does not follow exactly the opposite path of the original shape transition, but a third state appears. During flattening, the tubular hydrogel snaps rapidly and flattens after curling. Trigger conditions for gel plates are complex and time-consuming, and the snapping velocity, angle, and location of the sheet can be tuned by modulating the magnitude and location of stored energy within the hydrogel.

A pure humidity-responsive artificial flytrap leaf is shown in Figure 4d. It is obtained by bonding pre-stretched poly dimethyl siloxane (PDMS) layers prior to depositing electrospun polyethylene oxide nanofibers to induce hygroscopic bistability [62]. The moisture absorption capacity of the electrospun material is combined with the mechanical advantages of the preloaded structure to increase the actuating speed. When polyethylene oxide expands with increasing ambient humidity, its coupling with the passive layer causes the curvature of the artificial leaf to decrease until it snaps within 0.5 s. When the humidity is reduced, the initial state is restored.

### 4.3. Light-Driven Sensing and Actuation

**Figure 5:**
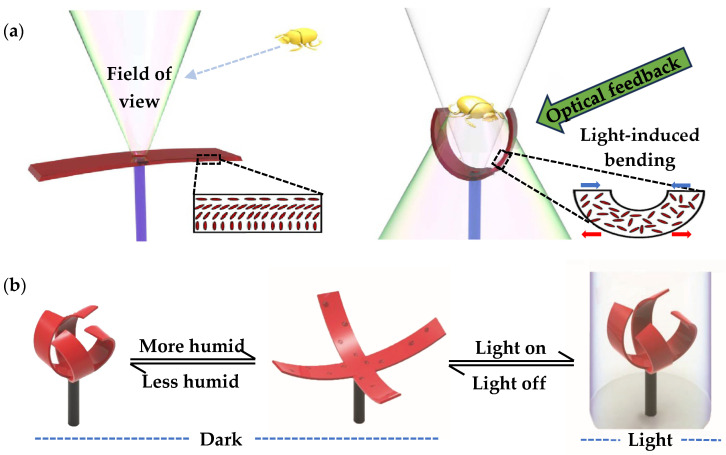
Light-driven smart material-based biomimetic flytrap structure, showing (**a**) a light-driven biomimetic flytrap leaf using a thin layer of light-responsive liquid crystal elastomer (LCE) as actuator [63]; (**b**) a liquid crystal network-based biomimetic flytrap structure, which can be controlled and modified by light and humidity [64].

Compared with the above hydrogel-based driving systems that can only perceive and react in a liquid environment, light-driven smart material has lower environmental requirements. Figure 5a shows a light-driven biomimetic flytrap structure using a thin layer of light-responsive liquid crystal elastomer (LCE) as an actuator [63]. The open-aligned LCE actuator is integrated with the fiber tip and leaves a window in the center for light injection. When the structure is subjected to light, the molecular alignment arrangement changes in LCE, sufficient optical feedback (reflected or scattered light) then generates strains, leading to expansion and shrinkage on different surfaces; the flytrap structure is then closed by the induced strain difference on the surfaces. Figure 5b shows a liquid crystal network-based biomimetic flytrap structure, which is controlled and modified by light and humidity [64]. It can be closed in low humidity levels and high light levels, and open in no light and high humidity levels. The sensing strategies of the light-driven structures are flexible and diverse, they are expected to be used in future adaptive and intelligent biomimetic structures.

### 4.4. Elastic Energy-Driven Rapid Snap-Trapping

The biomimetic strategies stated above mostly rely on the sensing capability to initiate snap-trapping. They lack elastic deformation-induced actuation, and the smart responses are usually in slow motion. Integration of the sensing capability into a bistable system may be more suitable for the biomechanics of the flytrap structure. The flytrap leaf is regarded as an elastic irregular curved shape. The macroscopic response of the flytrap after sensing the prey is that the leaves deform and pass through the energy barrier, and the stored elastic energy is instantly released and converted into kinetic energy, thereby forming a rapid snap-trapping action [65]. To date, the biomimetic structure close to this reaction is basically bistable. There are two ways to achieve the bistable characteristics of the biomimetic flytrap structure. These are not able to sense and are usually triggered by manual input. One strategy is to bind the bistable actuator between the artificial leaves, shape transitions between the two steady states of the actuator and drive the artificial leaves to morph. Although these structures have energy storage and release processes, they lack curvature changes as in a real leaf, and their reaction is similar to grasping rather than snap-trapping. The other strategy is to explore the curvature changes during the morphing of a bistable composite structure.

**Figure 6:**
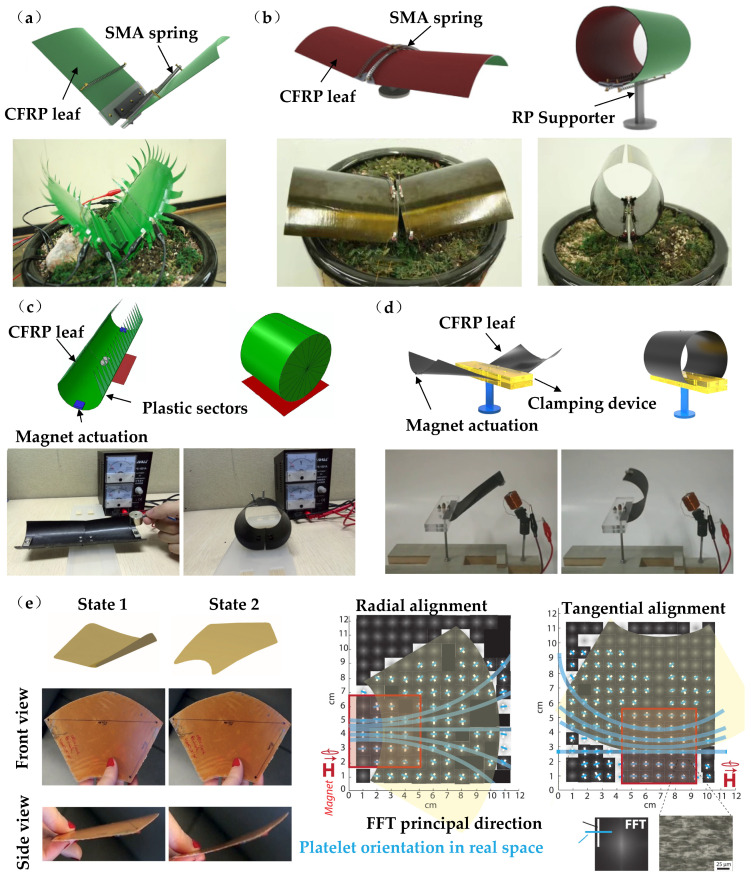
Elastic energy-driven bistable composite-based biomimetic flytrap structure, showing (**a**,**b**) OSB composite-based biomimetic flytrap structures actuacted by using shape memory alloys (SMAs), [66,67]; (**c**) an ESB-based biomimetic flytrap structure actuated by using magnets [69]; (**d**) an ESB-based flytrap structure with clamped boundaries actuated by using a clamping device [70]; (**e**) a magnet oriented particle-reinforced epoxy resin composite-based flytrap leaf structure [71].

Therefore, the morphing process of an OSB- or ESB-based bistable structure can be designed to introduce elastic energy-driven mechanisms into the flytrap structure. Figure 6a,b shows an OSB-based biomimetic flytrap structure, which is produced using asymmetric composite laminate, i.e., thermal residual stress-induced bistability, and actuated by SMA [66,67]. The bistable structure is used to imitate the artificial leaves, providing similar shapes to a real flytrap leaf. The two stable configurations correspond to the open and curled closed shapes of the real leaf with different structural curvatures. The SMA coil spring is used to induce snap-through of artificial leaves after being electrically heated, similar to the active motion of a flytrap, which is embedded on both surfaces of the artificial leaves to be repeatable of the rapid actions. Since the SMA-based actuator requires relaxation time to cool down, the morphing frequency of the structure is limited. It is verified by experiment that this type of bistable biomimetic flytrap structure is able to close within 100 ms, and the macroscopic rapid snapping deformation mechanism is similar to that of real flytrap leaves. The performance of the bistable biomimetic structure is then improved by designing the bistable characteristics of artificial leaves and changing the geometry and locations of the embedded SMA [68]. 

In addition, attempts have also been explored by using ESB-based structures, which also depict the real curvature changes of the flytrap leaves. Figure 6c shows an ESB-based biomimetic flytrap structure, actuated by using magnets. The bistability is derived by using the antisymmetric composite layup-induced geometry curvature effects [69]. On the upper side of an artificial leaf, the iron sheet is attached to the middle part of the outer curved edge to be attracted by magnets. The electromagnet is placed just above the iron patch in order to generate a suitable trigger force for the shape-changing activation. When the electromagnet is activated, the curve edge is subjected to magnetic force and produces a bending moment on the artificial leaves, which makes the biomimetic structure shift to the second stable state. This non-contact actuation simplifies the biomimetic structure and actuation design, with adjustable magnetic force through current control. A further improvement was then carried out to reduce the actuation force required to trigger the morphing action. Figure 4d shows an ESB-based flytrap structure with clamped boundaries [70]. The inner curved edges are clamped, and the snap-through of artificial leaves is constrained by a clamping device. Controlling the width of the clamping edge can effectively reduce the actuation force required to trigger the morphing actuation.

Although the shape-changing characteristics of the bistable artificial leaves are similar to those of the real flytrap leaves, the internal stress field of the real flytrap leaf is locally orthotropic and follows the geometric shape of the leaf as a whole, see details as discussed in Section 2. A further trial is shown in Figure 6e, where a magnet-oriented particle-reinforced epoxy resin composite was applied to mimic the internal stress field of a real flytrap leaf [71]. The microstructural changes are controlled by a magnetic field using locally oriented rigid anisotropic magnetic particles, in order to adjust the local prestrain and stiffness anisotropy of the composite. Compared with carbon fiber reinforced composites, the local residual strain of this biomimetic composite structure is more controllable, with a certain performance and shape gradient, and can achieve rapid shape-changing actuation, approaching the true shape transition mechanisms of the real flytrap.

**Figure 7:**
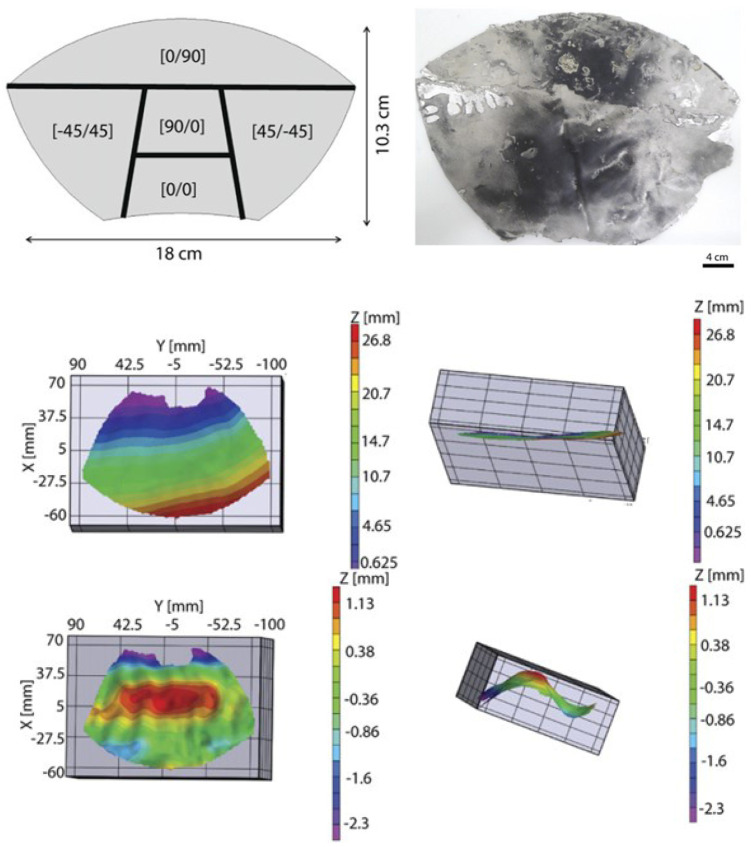
A multi-stimulus and multi-temporal responsive composites-based biomimetic flytrap structure, enabling fast snap-trapping through structural bistability and slow motion through diffusion processes using hydrogels [72].

A further strategy is considering multiple driving methods to mimic the biomechanics of the flytrap. Figure 7 shows a design scheme using multi-stimulus and multi-temporal responsive composites, enabling both fast morphing through structural bistability and slow morphing through diffusion processes using hydrogel [72]. The multi-responsive composites consist of a hydrogel layer and an architected particle-reinforced epoxy bilayer. The spatial distribution orientation of the magnetic responsive plates in each epoxy layer is achieved by using a magnetic field to induce in-plane mechanical properties and shrinkage. The epoxy double layer is used to adjust the prestress in the material, while the hydrogel layer controls the time response according to the hydration level. This smart composite-based biomimetic structure exhibits rapid and slow deformation in response to mechanical, magnetic, thermal and hydration stimuli. The integration of bistable and stimuli-responsive materials or smart materials can sense a variety of specific environmental conditions and react quickly.

## 5. Conclusions and Future Outlook

The biggest challenge for future smart composite bionics is to create a biomimetic flytrap structure that can pass the Turing test, which is designed to assess whether a model can accurately mimic the properties of a biological system. This means that a biomimetic structure is able to grow, and recover, and also has similar sense and response capabilities to that of a real Venus flytrap. Although some investigations have proposed self-growing robot concepts, the current biomimetic structures for flytraps still remain at the initial quickly growing stage. Therefore, this review focuses on the bionic strategies in terms of sensing, responding and actuation, as well as the rapid snap-trapping of the biomimetic Venus flytrap structures by employing the smart composite technology, aiming to enrich the diversities and reveal the fundamentals in order to further advance the multidisciplinary science and technological development into composite bionics.

The in-depth analysis of the biomechanics of a real Venus flytrap is helpful and essential in order to further promote and advance biomimetic structures. Flytrap leaves are not fully folded when stones, raindrops, etc., come into contact with trigger hairs, but only when prey is getting in contact. This energy-saving mechanism is still magical and worth further investigation. By using a multi-stable structure or driving control strategy, a multifunctional fly-catching biomimetic structure may be achieved. Compared with the enclosed trapping of the real flytrap, it is more realistic to mimic the bending or clamping motion for smart composite-based flytrap structures, and their driving strategies are diverse. The precision control of the express snapping and the load output range are still limited and depend on further development into smart composite technology. The sensing characteristics of IPMC are most similar to those of flytraps; whilst it lacks the elastic energy-derived rapid snapping. IPMC is promising to be applied as an actuator in the future owing to its sensing and fast response capabilities, in order to trigger the bistable artificial leaves. An exciting solution is a programmable microstructural bistable composite with multi-stimuli and multi-temporal responsive mechanics. The combination of bistability and bionics provides a new concept for developing the biomimetic structure with rapid shape morphing capability. In particular, the bistable composite structures offer similar shape-changing characteristics to a real flytrap leaf, and the elastic energy-driven actuation enables rapid capture motion. In the meantime, imitating the real curvature changes and internal stress field of a flytrap leaf improves the capture effectiveness of the biomimetic flytrap structure. 

## Data Availability

Data are available on request to the corresponding author.
